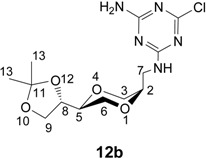# Synthesis of Novel Homo-*N*-Nucleoside Analogs Composed of a Homo-1,4-Dioxane Sugar Analog and Substituted 1,3,5-Triazine Base Equivalents

**DOI:** 10.3390/molecules13123092

**Published:** 2008-12-10

**Authors:** Qiang Yu, Dirk Schwidom, Alexander Exner, Per Carlsen

**Affiliations:** 1 Department of Chemistry, Norwegian University of Science and Technology (NTNU), N-7491 Trondheim, Norway; E-mail: qiang.yu@chem.ntnu.no (Q. Y.); 2 Socrates exchange student: Department of Chemistry, University of Hamburg, D-20146 Hamburg, Germany

**Keywords:** 1,3,5-Triazine, 1,4-Dioxane, Nucleoside analogue, Heterocycle, Homo-*N*-nucleoside, Nucleoside analogs

## Abstract

Enantioselective syntheses from dimethyl tartrate of 1,3,5-triazine homo-*N*-nucleoside analogs, containing a 1,4-dioxane moiety replacing the sugar unit in natural nucleosides, were accomplished. The triazine heterocycle in the nucleoside analogs was further substituted with combinations of NH_2_, OH and Cl in the 2,4-triazine positions.

## Introduction

Nucleoside analogs represent a potentially important class of medicinal active agents that have found uses in antitumor and antiviral drugs [1]. However, some drugs such as AZT [2,3], ddI [3], 3TC [4], are rapidly becoming less effective due to the developing drug resistance. Multi-drug resistance is a serious problem also for chemo- and anticancer therapy. In addition some drugs exhibit a variety of side effects. Therefore, it is desirable to develop new, active nucleoside analogs for application in medicine.

It is interesting to note that purines, amino derivatives of 1,3,5-triazines (also named *s*-triazines) and substituted guanines were found in the Orgueil meteorite [5,6]. The presence of *s*-triazines was interesting, since the various 1,3,5-triazines can be formed from hydrogen cyanide, ammonia and water, components believed to be plentiful in the primordial soup. Triazines may therefore have been abundant on early Earth [7]. The interesting question therefore arises as to what extend these triazines have played a role in the evolution of the original RNA [8].

The 1,3,5-triazine heterocyclic system is today found in a number of bioactive molecules such as herbicides and pharmaceutical products [9]. Various triazine substituted molecules exhibit diverse biological activities, having thus been reported as potentially cardiotonic [10,11], anti-HIV [12,13], antitumor [14] and anticancer agents [15]. Some triazines have found clinical applications. Hexamethylmelamine (HMM) has for example been used as antitumor agents [16]. Therefore nucleoside analogues containing triazines as the nucleic base equivalents are interesting subjects to study [17,18].

**Figure 1:**
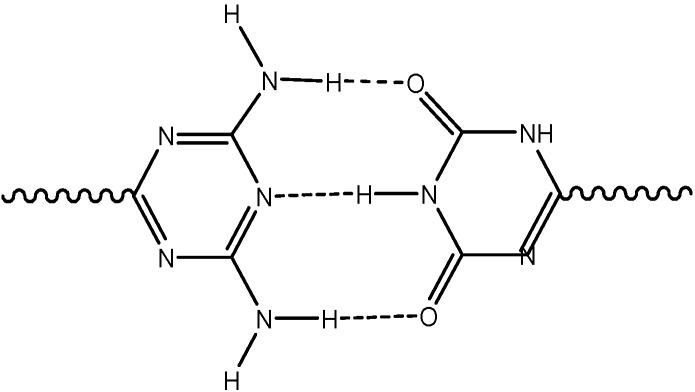
Interaction between NH_2_ and OH substituted triazines.

**Figure 2:**
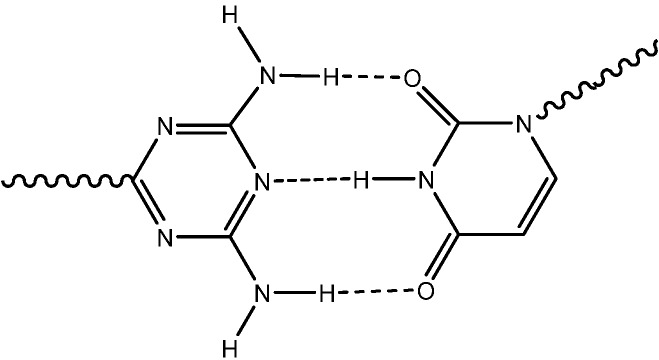
Interaction between triazine and uracil structures.

In the solid state 2-amino-4,6-dichloro-1,3,5-triazine form a ribbon structure of hydrogen bonded dimers[19]. Triazine oligomers were shown to self-assemble in duplex-strand structures [20]. Amino substituted triazine based oligomers and cyclic receptor molecules have also been studied [21]. Like the natural complementary pyrimidine and purine nucleic bases forming associates through hydrogen bonds as observed in the nucleic acid structures, amino and hydroxyl substituted triazines can self-assemble into supramolecular structures through the formation of hydrogen bonds [22,23]. A typical example of such associates is sketched in Figure 1. In addition, it has been observed that triazine derivatives, for example 2,6-diamino-1,3,5-triazines, may function as purine mimics and can recognize pyrimidines, forming associates with for example uracils or thymines through hydrogen bonding [24,25,26], Figure 2.

**Figure 3:**
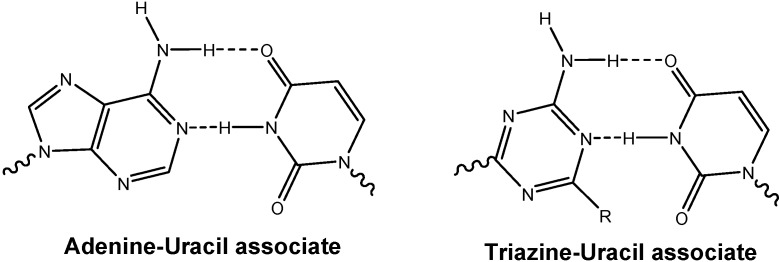
Interaction between nucleic bases (adenine-uracil) and monoaminotriazine derivatives with uracil.

Monoaminotriazine nucleoside analogs also represent interesting structures, as they may mimic adenines, forming associates with uracil analogs, Figure 3.

These observations suggest the use of triazine nucleoside analogs as leads for the development of new nucleoside and nucleotide analogs for medicinal application. Considering the potential applications of the 1,3,5-triazine based systems, surprisingly few triazine nucleoside analogs or triazine glycosides have been reported in the literature [27,28,29,30,31]. For these reasons we found it viable to initial a study of the synthesis of triazine based nucleoside analogs.

## Results and Discussion

**Figure 4:**
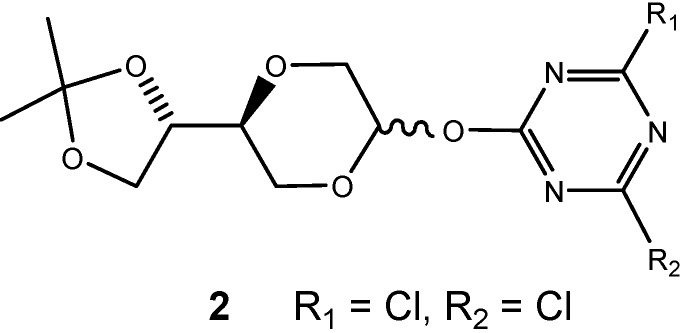
Triazine nucleoside analogs.

Rather than applying the five-membered sugars found in the natural nucleosides, we targeted on the application of the more robust and more flexible sugar analogs containing the 1,4-dioxane moiety, for example compound **1**, whose synthesis has been reported in a previous publication [32]. Compound **1**, containing an unnatural sugar analog may also prove to be robust in biologic systems, as enzymes may not find pathways to convert the unnatural nucleoside system. The synthesis of the triazine *O*-nucleoside analogs **2**, in which a 1,4-dioxane sugar moiety was connected to a dichloro substituted triazine ring system via an ether linkage at the anomeric position (Figure 4) was thus the initial synthetic goal.

**Scheme 1:**
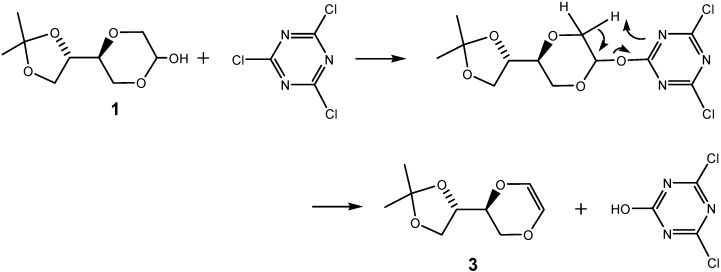
Reaction of 1,4-dioxane sugar analog **1** with 2,4,6-trichlorotriazine.

Preparation of this structure was attempted from the 1,4-dioxane sugar analog **1**, by its reaction with 2,4,6-trichloritriazine. However, all attempts failed to give the desired product, as formation of the elimination product **3** always predominated (Scheme 1). This was surprising, as triazine glycosides are known [30]. However, in the 1,4-dioxane system, the CH_2_-group neighboring the anomeric -OH group may here actually facilitate an elimination reaction pathway, providing instead product **3**, which was isolated and the structure confirmed by NMR spectroscopy. As a spin-off of this observation, we are currently exploring the possible use of chlorotriazine as an elimination reagent for the formation of alkenes from alcohols.

**Figure 5:**
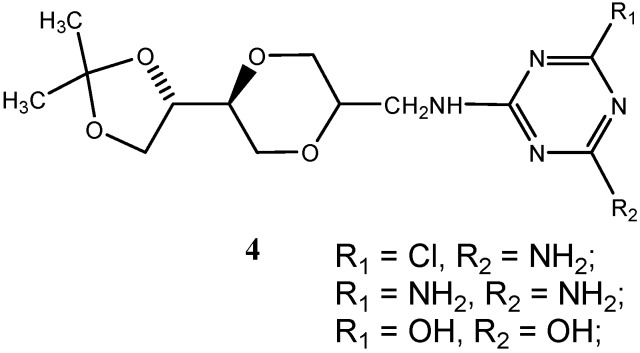
Structure of triazine homo-*N*-nucleoside analogs.

**Scheme 2:**
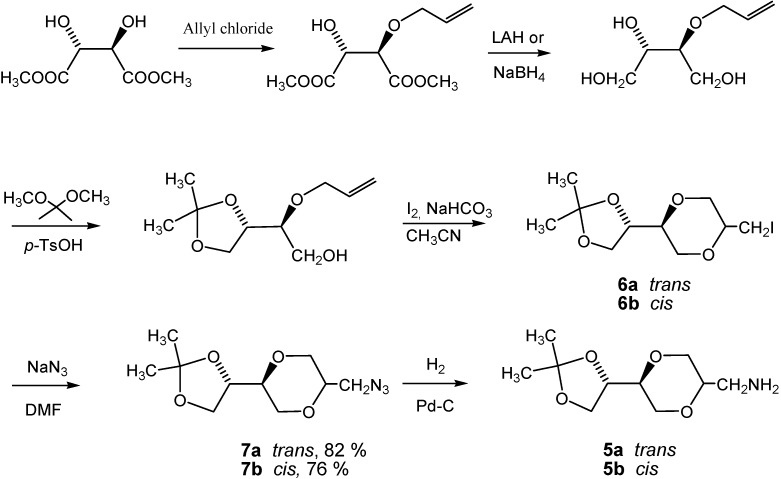
Synthesis of homo amino-1,4-dioxane *pseudo*-sugar **5a** and **5b**.

The observed instability of the triazine *O*-nucleoside analog **2** (Scheme 1), prompted us to design the potentially more stable triazine homo-*N*-nucleoside analogs, **4** (Figure 5). *N*-glycosidic nucleoside analogs represent a known, well established class of stable modified nucleosides [33,34,35,36,37]. For increased stability, the linking ether group was thus replaced by the corresponding –CH_2_-NH- linker. This was expected to result in a conformationally more flexible but also a chemically and biologically more stable structure. Hence, the alternative structures **4**, became the new targets. Nucleophilic, aromatic substitution readily takes place with chlorosubstituted triazines. Therefore, we could conveniently adopt a general synthetic procedure in which variously substituted chlorotriazines were reacted with for example the amino sugar analogues **5** (Scheme 2).

For the purpose of constructing triazine nucleoside analogs that may function as complementary bases to the naturally occurring bases, controlled substitution in the 2- and 4-positions of the triazine heterocyclic system was investigated. Thus, the triazine may be substituted with various combinations of H, OH and NH_2_ groups. The combinations that included H were not prepared, but instead chloro substituted triazines were used. Chloro-substituted purine and pyrimidine systems have also found uses in medicinal chemistry. The OH- / NH_2_ substituted triazine may correspond to the nucleobases cytosine and guanine, while the OH / OH combination corresponds to uracil and thymine. It has previously been reported that such triazine systems may form hydrogen bond with the complementary natural bases [38].

The enantiomerically pure homo amino sugar analogs **5a** and **5b** were readily prepared from iodides **6a** and **6b** by the reaction with sodium azide in DMF [39,40], to give azides **7a** and **7b** in 76 and 82 % yields, respectively. Subsequent hydrogenolysis of the azides using Pd-C as the catalyst readily provided the corresponding amines **5a** and **5b**, respectively, in essentially quantitative yields. Iodides **6** were obtained from (2*R*,3*R*)-dimethyl tartrate as previously reported [41] according to the reaction sequence shown in Scheme 2. Product **6** was isolated as an approximately 1:1 diastereomeric mixture of *trans*-**6a** and *cis*-**6b**. The application of the tartrates as starting materials, readily available from the chiral pool, conveniently allow for the synthesis of all the possible stereoisomers of the target molecules.

**Figure 6:**
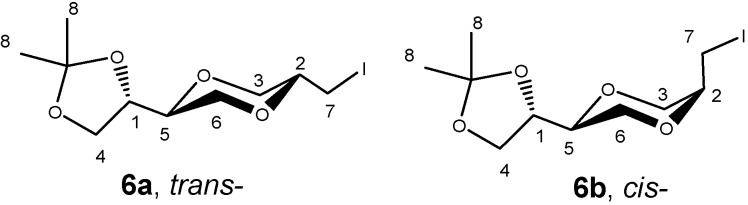
Molecular structures **6a** and **6b**.

Structures **6a** and **6b** were established by NMR spectroscopy. Thus, product **6a** exhibited a large H-2/H-3 coupling constant (10.2 Hz), which was in agreement with an axial H-2 proton. The corresponding coupling constant (3.6 Hz) observed for compound **6b** was in agreement with the corresponding *cis*-isomer configuration (Figure 6).

**Scheme 3:**
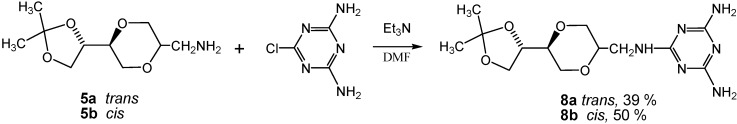
Synthesis of diaminotriazine homo-*N*-nucleoside analogues **8a** and **8b**.

The 3,5-diaminotriazine homo-*N*-nucleoside analogs **8a** and **8b** were prepared by the reaction of the amino sugar analogs **5a** and **5b** respectively with 2-chloro-4,6-diaminotriazine in DMF in the presence of triethylamine. Thus, *trans*-amine **5a** afforded the desired diamino triazine homo-*N*-nucleoside analog **8a** in 39 % yield. The corresponding *cis-*triazine homo-*N*-nucleoside **8b** was obtained in 50 % isolated yield (Scheme 3).

**Scheme 4:**
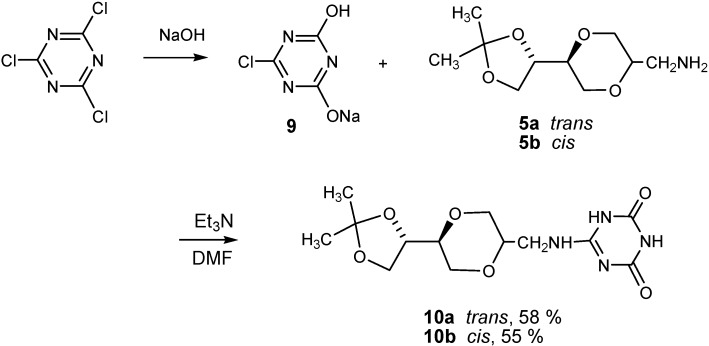
Synthesis of triazine homo-*N*-nucleoside analogue **10a** and **10b**.

For the purpose of mimicking the thymine and uracil bases, the 2,4-dihydroxy substituted triazine systems were also prepared. The 2-chloro-4,6-dihydroxy-1,3,5-triazine was readily prepared according to a known procedure [42] from commercial available 2,4,6-trichlorotriazine by treatment in an aqueous sodium hydroxide solution. This provided the monosodium salt of 2-chloro-4,6-dihydroxy-1,3,5-triazine **9**, which was further coupled with **5a** in DMF in the presence of triethylamine to provide triazine nucleoside analogue **10a** in 58 % isolated yield. The *cis-* triazine nucleoside analogue **10b** was similarly prepared from **5b** in 55 % isolated yield (Scheme 4).

**Scheme 5:**
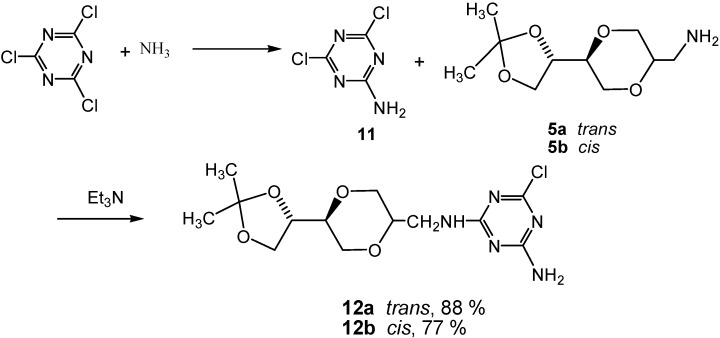
Synthesis 2-chloro-4-amino triazine homo-*N*-nucleoside analogues **12a** and **12b**.

Monoaminotriazine nucleoside analogues were also of interest, as they may mimic adenines, forming associates with for example uracil analogs (Figure 3). Thus for further structural variations of the triazine *N*-nucleoside analogs, **5a** and **5b**, respectively, were coupled with 2,4-dichloro-6-aminotriazine (**11**), which was obtained from 2,4,6-trichlorotriazine by treatment with ammonia at 0 ^o^C [43]. Products **12a** and **12b** were obtained in 88 % and 77 % isolated yields (Scheme 5).

Interestingly, a chromatographically pure sample of **12a** was observed to exhibit an exchange effects in the ^1^H-NMR spectra. When the ^1^H-NMR of **12a** was recorded at 25 ^o^C in CDCl_3_, the spectra exhibited the presence of two series of signals ascribed to two different structures in a 5:8 ratio. However, these signals collapsed into a single set of signals when the temperature of the sample exceeded 50 ^o^C. In the course of this process, the chemical shifts also changed. Product **12b** exhibited a similar behavior.

The observed temperature effects on the NMR spectra of **12** is not yet clear, but may be rationalized in terms of dimerization of **12** or due to the presence of rotamers. Aminotriazines may form complexes through hydrogen bond formation. In this respect it is also worthwhile to note that a number of possible tautomers of the aminotriazine system may play a role in for example dimer formation. Triazine systems have been observed to exhibit rotational isomerism [44,45,46,47]. For the present system, **12**, there are several rotatable bonds. One of these, the triazine-NH bond, may be associated with a high rotational barrier, for example due to a possible, partial C-N double bond character, as the result of tautomer formation. An initial, though rudimentary conformational analysis, using theoretical molecular mechanics calculations (ChemModel/MMX), indicated the presence of rotamers and several low-energy conformations. Due to a number of possible rotatable bonds and the dimensional limitations associated with the applied method, we can not here point to distinct structures related to the transformations observed in the NMR experiments. These aspects of the properties of products **12a** and **12b** have so far not been further pursued.

## Conclusions

In conclusion, optically active homo-*N*-nucleoside analogs containing a 1,3,5-triazine base equivalent, were synthesized from dimethyl tartrate. The triazines, including the 2,4-diamino substituted, the dihydroxy substituted and chloro-amino 2,4-disubstituted triazines, were linked to the homo-1,4-dioxoane sugar analog moiety via a CH_2_NH linker. Biologic screening of the prepared nucleoside analogs is now in progress.

## Experimental

### General

NMR spectra were recorded on Bruker Avance DPX 400 instruments. Chemical shifts are reported in ppm using TMS as the internal standard in CDCl_3_ or relative to 2.50 ppm for ^1^H and 39.99 ppm for ^13^C in DMSO-*d*_6_ or 3.31 ppm for ^1^H and 49.15 ppm for ^13^C in CD_3_OD. Structural assignments were based on ^1^H, ^13^C, DEPT135 and 2D spetra, COSY, HSQC, HMBC, NOESY. EI-Mass and ESI spectra were recorded on a Finnigan MAT 95XL spectrometer. IR spectra of the solid products were obtained on a Thermo Nicolet FT-IR Nexus spectrometer equipped with a Smart Endurance reflection cell. Silica gel Kieselgel 60G (Merck) was used for Flash Chromatography. The solvents were purified by standard methods. The preparations of **6a** and **6b** were described in the previous paper [41].

*(5S)-5-[(4S)-2,2-dimethyl-1,3-dioxolan-4-yl]-6-dihydro-1,4-dioxane* (**3**). To compound **1** (0.66 g, 3.2 mmol) in dry THF (12 mL) was added sodium hydride (101 mg, 4.2 mmol) and the mixture was stirred for 10 min. at room temperature. 2,4,6-Trichloro-1,3,5-triazine (0.80 g, 6.2 mmol) was then added to the mixture, which was refluxed for 4 hours. The mixture was concentrated and purified by flash chromatography by using a mixture of diethyl ether and *n*-hexane (1:3) as eluent to provide product **3** (0.1 g, 17 %). ^1^H-NMR (400 MHz, CDCl_3_) δ 1.38 (d, *J* = 0.4 Hz, 3H, H-7), 1.45 (d, *J* = 0.4 Hz, 3H, H-7), 3.84 (dd, *J* = 11.2 Hz, 7.2 Hz, 1H, H-3), 3.89 (dd, *J* = 8.4 Hz, 6.8 Hz, 1H, H-6), 3.96-4.0 (m, 1H, H-4), 4.04-4.10 (m, 2H, H-3 and H-6), 4.27 (dt, *J* = 5.2 Hz, 6.8 Hz, 1H, H-5), 5.99 (d, *J* = 3.4H, 1H, H-2), 6.04 (d, *J* = 3.4 Hz, 1H, H-1) ppm. ^13^C-NMR (100 MHz, CDCl_3_) δ 25.3, 26.2, 65.0, 65.3, 73.3, 74.3, 109.9, 126.5, 126.8 ppm.

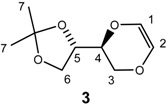


*(2S,5S)-2-[(4S)-2,2-dimethyl-1,3-dioxolan-4-yl]- 5-azidomethyl-1,4-dioxane* (**7a**). To the solution of **6a** (1.30 g, 4 mmol) in DMF, sodium azide (1.41 g, 22 mmol) was added. The mixture was stirred overnight at 80 °C. The mixture was concentrated under reduced pressure and the residue was extracted with chloroform. The filtrate was concentrated and the residue purified by flash chromatography using a mixture of ethyl ether and *n*-hexane (1:2) as eluent to give product **7a** (yield 0.79, 82 %). ^1^H-NMR (400 MHz, CDCl_3_) δ 4.06 (dt, 1H, *J* = 6.8 Hz, 5.6 Hz, H-8), 3.97 (dd, 1H, *J* = 8.4 Hz, 6.0 Hz, H-9 eq), 3.85 (dd, 1H, *J* = 11.6 Hz, 2.4 Hz, H-3 eq), 3.79 (dd, 1H, *J* = 8.4 Hz, 7.2 Hz, H-9 ax), 3.77 (dd, 1H, *J* = 11.2 Hz, 2.4 Hz, H-6 eq), 3.76-3.69 (m, 1H, H-2), 3.61 (ddd, 1H, *J* = 2.0 Hz = 5.2 Hz, H-5), 3.53 (dd, 1H, *J* = 11.2 Hz, 7.6 Hz, H-6 ax), 3.48 (dd, 1H, *J* = 11.6 Hz, 10.4 Hz, H-3 ax), 3.26 (d, 2H, *J* = 5.2 Hz, H-7), 1.43 (d, 3H, *J* = 0.4 Hz, H-13), 1.36 (d, 3H, *J* = 0.4 Hz, H-13). ^13^C-NMR (100 MHz, CDCl_3_) δ 109.9, 75.5, 75.1, 74.0, 68.6, 67.5, 65.2, 51.6, 26.4, 25.4. IR (neat): 2984, 2866, 2096, 1448, 1370, 1292 cm^-1^. HRMS (*m/z*, ESI): for C_10_H_17_N_3_O_4_ [M+Na]^+^; Calcd. 266.1116; Found 266.1108.

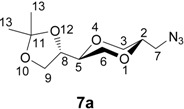


*(2S,5R)-2-[(4S)-2,2-dimethyl-1,3-dioxolan-4-yl]- 5-azidomethyl-1,4-dioxane* (**7b**). The *trans*- compound **7b **was prepared by following the same procedure as described for **7a**. Product **7b** was obtained as a colorless oil (0.73 g, 75.8 %). ^1^H-NMR (400 MHz, CDCl_3_) δ 4.34 (dd, 1H, *J* = 13.6 Hz, 6.8 Hz, H-8), 4.04 (dd, 1H, *J* = 8.4 Hz, 6.4 Hz, H-3 eq), 3.82-3.76 (m, 3H, H-2, H-6), 3.75 (dd, 1H, *J* = 8.4 Hz, 6.8 Hz, H-3 ax), 3.70 (dd, 1H, *J* = 6.8 Hz, 12 Hz, H-9 ax), 3.69-3.61 (m, 1H, H-7), 3.66 (dd, 1H, *J* = 6.8 Hz, J = 1.6 Hz, H-9 eq), 3.63 (dd, 1H, *J* = 3.2 Hz, *J* = 6.4 Hz, H-5), 3.37-3.32 (m, 1 H, H-7), 1.44 (d, 3H, *J* = 0.4 Hz, H-13), 1.38 (d, 3H, *J* = 0.4 Hz, H-13) ppm. ^13^C-NMR (100 MHz, CDCl_3_) δ 110.0, 74.9, 74.0, 71.9, 65.8, 65.3, 63.2, 45.0, 26.6, 25.6 ppm. HRMS (*m/z,* ESI): for C_10_H_17_N_3_O_4_ (M+Na^+^), Calcd. 266.1116, found 266.1115.

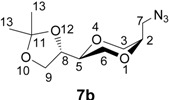


*(2S,5S)-2-[(4S)-2,2-dimethyl-1,3-dioxolan-4-yl]-5-aminomethyl-1,4-dioxane* (**5a**). To compound **7a **(90.1 mg, 0.365 mmol) dissolved in MeOH (5 mL) was added Pd-C (5 % Pd, 13.4 mg, 0.2 mol %). The suspension was stirred for 2.5 hours under a hydrogen atmosphere (1.8 – 2.0 bar), until TLC indicated complete conversion. The catalyst was removed by filtration and the filtrate was concentrated to dryness. The resulting residue was dissolved in 1 mL MeOH, and filtered through a plug of Celite, which was then washed five times with MeOH (2 Ml). The combined filtrates were evaporated to give a colorless oil as product. The yield was 80.4 mg (0.371 mmol, 99 %) product. It became solid after standing a couple of days. The ^1^H-NMR (400 MHz, CDCl_3_) δ 4.06 (dt, 1H, *J* = 6.8 Hz, 6.4 Hz, H-8), 3.97 (dd, 1H, *J* = 8.4 Hz, 6.8 Hz, H-9eq), 3.85 (dd, 1H, *J* = 11.6 Hz, 2.4 Hz, H-3 eq), 3.79 (dd, 1H, 8.0 Hz, 7.2 Hz, H-9 ax), 3.74 (dd, 1H, *J* = 10.8 Hz, 2.0 Hz, H-6 eq), 3.62-3.55 (m, 2H, H-2, H-5), 3.52 (dd, 1H, *J* = 10.8 Hz, 10.4 Hz, H-6 ax), 3.41 (dd, 1H, *J* = 11.6 Hz, 10.4 Hz, H-3 ax), 2.78 (dd, 1H, *J* = 13.2 Hz, 4.0 Hz, H-7), 2.69 (dd, 1H, *J* = 13.2 Hz, 8.4 Hz, H-7), 2.57 (broad s, 2H, NH_2_), 1.43 (d, 3H, *J* = 0.4 Hz, H-13), 1.36 (d, 3H, *J* = 0.4 Hz, H-13) ppm. ^13^C-NMR (100 MHz, CDCl_3_) δ 109.7, 75.9, 75.6, 75.2, 69.1, 67.4, 65.2, 42.7, 26.4, 25.4 ppm. HRMS (*m/z*, ESI): for C_10_H_17_N_3_O_4_ (M+H^+^), calcd. 218.1392, found 218.1389.

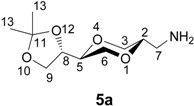


*(2S,5R)-2-[(4S)-2,2-dimethyl-1,3-dioxolan-4-yl]-5-aminomethyl-1,4-dioxane* (**5b**). The *cis*- compound **5b** was prepared by following the same method as for synthesis of **5a**. ^1^H-NMR (400 MHz, CDCl_3_) δ 4.38 (ddd, 1H, *J* = 6.8 Hz, 6.8 Hz, 6.8 Hz, H-8), 4.04 (dd, 1H, *J* = 8.4 Hz, 6.8 Hz, H-9 eq), 3.76 (d, 2H, *J* = 4.4 Hz, H-3), 3.73 (dd, 1H, *J* = 8.0 Hz, *J* = 7.2 Hz, H-5), 3.68-3.58 (m, 4 H, H-9 ax, H-6, H-2), 3.10 (dd, 1H, *J* = 13.2 Hz, 8.8 Hz, H-7), 2.79 (dd, 1H, *J* = 13.2 Hz, 4.4 Hz, H-7), 2.42 (bs, 2H, NH_2_), 1.44 (d, 3H, *J* = 0.4 Hz, H-13), 1.38 (d, 3H, *J* = 0.4 Hz, H-13) ppm. ^13^C-NMR (100 MHz, CDCl_3_): δ 109.8, 74.6, 74.4, 73.9, 65.8, 65.5, 63.2, 41.1, 26.6, 25.5 ppm. IR (neat): 2990, 2864, 1625, 1450, 1434, 1376, 1367, 1122, 1022, 979 cm^-1^. HRMS (*m/z*, ESI): for C_10_H_17_N_3_O_4_ (M+H^+^), calcd. 218.1392, found 218.1394.

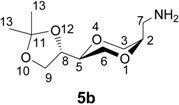


*2,4-Diamino-6-[[(2S,5S)-2-[(4S)-2,2-dimethyl-1,3-dioxolan-4-yl]-1,4-dioxan-5-yl]methylamino]-1,3,5- triazine* (**8a**). To a solution of **5a** (244 mg, 1.12 mmol) and triethylamine (0.16 mL, 1.15 mmol) in DMF (16 mL) was added 2-chloro-4,6-diaminotriazine (80 mg, 0.55 mmol). The mixture was heated at 80 ^o^C and stirred overnight. The solution was evaporated under high vacuum and the residue was purified by column chromatography using gradient eluent systems [dichloromethane and methanol (20:1); dichloromethane and methanol (10:1)]. Product **8a** was obtained as a white solid in 39 % yield. ^1^H-NMR (400 MHz, CDCl_3_) δ 6.40 (t, 1H, *J* = 6 Hz, NH), 6.06 (s, 2H, NH_2_), 5.95 (s, 2H, NH_2_), 3.96-4.01 (m, 1H, H-8), 3.89 (dd, 1H, *J* = 8.2 Hz, 6.6 Hz, H-9), 3.79 (dd, 1H, *J* = 11.6, 2.6 Hz, H-3), 3.72 (dd, 1H, *J* = 8.2 Hz, 7.0 Hz, H-9), 3.67 (dd, 1H, *J* = 11.2 Hz, 2 Hz, H-6), 3.52-3.58 (m, 1H, H-2), 3.44-3.48 (m, 1H, H-5), 3.30-3.37 (m, 1H, H-6), 3.21-3.29 (m, 2H, H-3, H-7), 3.08-3.15 (m, 1H, H-3), 1.30 (s, 3H, H-13), 1.24 (s, 3H, H-13) ppm. ^13^C-NMR (100 MHz, CDCl_3_) δ 167.0, 166.4, 108.4, 74.5, 73.6, 69.1, 66.6, 64.3, 41.0, 26.1, 25.3 ppm. IR (neat): 3353, 3130, 2983, 1633, 1608, 1537, 1455, 1108, 1048 cm^-1^. HRMS (*m/z*, ESI): for C_13_H_22_N_6_O_4_ (M+H^+^), calcd. 327.1780, found 327.1780.

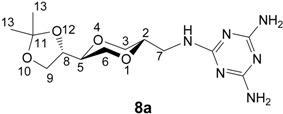


*2,4-Diamino-6-[[(2S,5R)-2-[(4S)-2,2-dimethyl-1,3-dioxolan-4-yl]-1,4-dioxan-5-yl]-methylamino]-1,3,5-triazine* (**8b**). *cis*-Compound **8b** was prepared in 50 % yield from **5b** by following the same method as for the synthesis of **8a**. ^1^H-NMR (400 MHz, CDCl_3_) δ 6.34 (t, 1H, *J* = 6Hz, NH), 5.43 (s, 4H, NH_2_), 4.35 (ddd, 1H, *J* = 6.8Hz, 6.8 Hz, 6.8 Hz, H-8), 4.01 (dd, 1H, *J* = 8.4 Hz, 6.4 Hz, H-9), 3.59-3.82 (m, 7H, H-9, H-2, H-3, H-5, H-6), 3.47-3.54 (m, 2H, H-7), 1.43 (s, 3H, H-13), 1.37 (s, 3H, H-13) ppm. ^13^C-NMR (100 MHz, CDCl_3_) δ 167.5, 167.2, 166.8, 109.9, 74.5, 74.0, 72.1, 65.8, 65.3, 63.2, 40.0, 26.6, 25.4 ppm. IR (neat) 3342, 3168, 2983, 1539, 1449, 1122, 1065 cm^-1^. HRMS (*m/z*, ESI): for C_13_H_22_N_6_O_4_ (M+H^+^), calcd. 327.1780, found 327.1787.

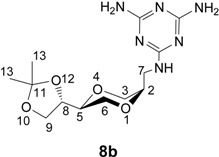


*2,4-Dihydroxy-6-[[(2S,5S)-2-[(4S)-2,2-dimethyl-1,3-dioxolan-4-yl]-1,4-dioxan-5-yl]methylamino]-1,3,5-triazine* (**10a**). To compound **5a** (88 mg, 0.41 mmol) and triethylamine (0.08 mL, 0.58 mmol) in DMF (10 mL) was added the monosodium salt of 2-chloro-4,6-dihydroxy-1,3,5-triazine (**9**, 127 mg, 0.62 mmol). The mixture was heated at 80 ^o^C and stirred overnight. The solvent was evaporated under high vacuum and the residue was purified by flash chromatography using a mixture of dichloromethane and methanol (10:1) as eluent. The product **10a** (67 mg) was obtained in 43 % yield. ^1^H-NMR (400 MHz, CDCl_3_) δ 10.40 (broad s, 2H, OH), 7.02 (s, 1H, NH), 4.00 (ddd, 1H, *J* = 6.8 Hz, 6.8 Hz, 6.8 Hz, H-8), 3.90 (dd, 1H, *J* = 8.2 Hz, 6.6 Hz, H-9), 3.80 (dd, 1H, *J* = 11.6 Hz, 2.4 Hz, H-3), 3.71-3.76 (m, 2H, H-9, H-6), 3.57-3.61 (m, 1H, H-2), 3.46-3.50 (m, 1H, H-5), 3.17-3.44 (m, 4H, H-6, H-3, H-7), 1.30 (s, 3H, H-13), 1.24 (s, 3H, H-13) ppm. ^13^C-NMR (100 MHz, CDCl_3_) δ 156.5, 108.5, 74.4 (C-5 and C-8), 72.8, 68.1, 66.7, 64.3, 40.9, 26.1, 25.3 ppm. IR (neat) 3090, 2983, 1720, 1628, 1490, 1131, 1061 cm^-1^. HRMS (*m/z*, ESI), for C_13_H_20_N_4_O_6_ (M+H^+^), calcd. 329.1461, found 329.1469.

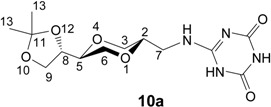


*2,4-Dihydroxy-6-[[(2S,5R)-2-[(4S)-2,2-dimethyl-1,3-dioxolan-4-yl]-1,4-dioxan-5-yl]-methylamino]-1,3,5-triazine* **(10b**). *cis*-Compound **10b** was prepared from **5b** by following the same method as for the synthesis of **10a**. ^1^H-NMR (400 MHz, CDCl_3_) δ 10.62 (broad s, 1H, OH), 10.48 (broad s, 1H, OH), 6.90 (s, 1H, NH), 4.24 (ddd, 1H, *J* = 6.4 Hz, 6.4 Hz, 6.4 Hz, H-8), 3.94 (dd, 1H, *J* = 8.4 Hz, 6.8 Hz, H-9), 3.72 (dd, 1H, *J* = 8 Hz, 6.8 Hz, H-9), 3.48-3.69 (m, 7H, H-5, H-6, H-3, H-2, H-7), 3.36-3.42 (m, 1H, H-7), 1.31 (s, 3H, H-13), 1.27 (s, 3H, H-13) ppm. ^13^C-NMR (100 MHz, CDCl_3_) δ 156.3, 149.9, 108.5, 73.8, 73.5, 70.1, 64.8, 64.7, 61.9, 39.3, 26.4, 25.3 ppm. IR (neat) 3126, 2985, 1741, 1588, 1121, 1060 cm^-1^. HRMS (*m/z*, ESI), for C_13_H_20_N_4_O_6_ (M+H^+^), calcd. 329.1461, found 329.1462.

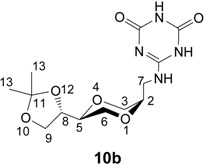


*2-Chloro-4-amino-6-[[(2S,5S)-2-[(4S)-2,2-dimethyl-1,3-dioxolan-4-yl]-1,4-dioxan-5-yl]-methyl-amino]-1,3,5-triazine* (**12a**). To compound **5a** (40 mg, 0.18 mmol) and triethylamine (0.04 mL, 0.29 mmol) in DMF (6 mL) was added 2,4-dichloro-6-amino-1,3,5-triazine (**11**, 31 mg, 0.19 mmol). The mixture was stirred at room temperature overnight. The DMF was evaporated under high vacuum and the residue was purified by flash chromatography using a mixture of dichloromethane and methanol (20:1) as the eluent. The product (53 mg) was obtained in 88 % yield. ^1^H-NMR (400 MHz, CDCl_3_, 25 ^o^C) δ 1.36, 1.43 (s, 2×CH_3_ for both rotamers), 3.25-3.32 (m), 3.37-3.46 (m), 3.48-3.61 (m), 3.65-3.82 (m), 3.86-3.93 (m), 4.03-4.08 (m), 5.20-5.40 (broad, 2H, NH_2_, for both rotamers), 5.83 (t, NH), 6.14 (t, NH) ppm. ^1^H-NMR (400 MHz, CDCl_3_, 50^o^C) δ 5.71 (broad s, 1H, NH), 5.19 (broad s, 2H, NH_2_), 4.03-4.08 (m, 1H), 3.95 (dd, 8.2, *J* = 6.6 Hz, 1H), 3.85-3.88 (m, 1H), 3.81 (dd, *J* = 8.2, *J* = 7.0 Hz, 1H, ), 3.74 (dd, *J* =10.6 and *J* = 1.4 Hz, 1H), 3.65-3.71 (m, 1H), 3.48-3.60 (m, 3H), 3.40 (dd, *J* = 11.4 and 10.6 Hz, 1H), 3.26-3.32 (m, 1H), 1.413, 1.412, 1.342, 1.341 (6H, 2×CH_3_) ppm. ^13^C-NMR (100 MHz, CDCl_3_, 50 ^o^C) δ 25.3, 26.3, 41.8, 65.1, 67.5, 68.9, 73.6, 75.1, 75.3, 109.8, 166.5, 167.3, 169.8. IR (neat) 3392, 3265, 3119, 2983, 1655, 1576, 1485, 1103, 1056 cm^-1^. HRMS (*m/z*, ESI), for C_13_H_20_ClN_5_O_4_ (M+H^+^), calcd. 346.1282, found 346.1283.

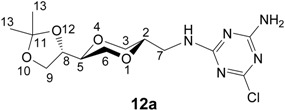


*2-Chloro-4-amino-6-[[(2S,5R)-2-[(4S)-2,2-dimethyl-1,3-dioxolan-4-yl]-1,4-dioxan-5-yl]-methyl-amino]-1,3,5-triazine* (**12b**) *cis*-Compound **12b** was prepared by following the same method as for synthesis of **12a**. ^1^H-NMR (400 MHz, CDCl_3_) δ 1.37, 1.44 (s, 2×CH_3_ for both rotamers), 3.49-3.55(m), 3.57-3.67(m), 3.75-3.86(m), 3.99-4.06 (m, 1H, for both rotamers), 4.31-4.36 (m, 1H, for both rotamers), 5.3-5.9 (broad, 2H, NH_2_, for both rotamers), 6.02, 6.11 (1H, NH, for both rotamers) ppm. ^13^C-NMR (100 MHz, CDCl_3_) δ 25.2, 25.3, 26.4, 26.5, 62.6, 62.9, 65.4, 65.48, 65.52, 65.6, 70.9, 71.3, 74.0, 74.4, 166.1, 166.3, 166.6, 167.0, 169.2 ppm. IR (neat) 3327, 3270, 3171, 2983, 1646, 1563, 1482, 1123, 1065 cm^-1^. HRMS (*m/z*, ESI), for C_13_H_20_ClN_5_O_4_ (M+H^+^), calcd. 346.1282, found 346.1295.